# Digital 3D Technology in Pediatric Surgical Oncology: An Expert White Paper

**DOI:** 10.3390/cancers18193117

**Published:** 2026-09-25

**Authors:** Nick de Groot, Pamela Lustig, Sadie Lynn Love, Chris Goode, Joseph Fusco, Andrew Davidoff, Alida van der Steeg, Lucas Krauel, Zachary Abramson, Matthijs Fitski

**Affiliations:** 1 Princess Máxima Center for Pediatric Oncology, 3584 CS Utrecht, The Netherlands; 2 3D Unit (3D4H), SJD Barcelona Children’s Hospital, 08950 Barcelona, Spain; 3 Department of Radiology, St Jude Children’s Research Hospital, Memphis, TN 38105, USA; 4 Department of Pediatric Surgery, Monroe Carell Jr. Children’s Hospital at Vanderbilt, Nashville, TN 37232, USA; joseph.fusco@vumc.org; 5 Department of Surgery, St. Jude Children’s Research Hospital, Memphis, TN 38105, USA; andrew.davidoff@stjude.org; 6 Pediatric Surgical Oncology Unit, Department of Pediatric Surgery, PCCB-SJD Barcelona Children’s Hospital, 08950 Barcelona, Spain

**Keywords:** pediatric surgical oncology, three-dimensional models, preoperative planning, surgical navigation, patient education and simulation

## Abstract

Digital three-dimensional (3D) visualization is an evolving field that is being introduced into pediatric surgical oncology, where it addresses challenges related to complex anatomy and difficulties accurately defining surgical margins. This paper provides an overview of digital 3D modeling in pediatric surgical oncology and explores ways to further advance this field. Digital 3D models may improve various aspects of surgery, such as preoperative planning, intraoperative guidance, patient education, and surgical training. Traditionally, 3D models are manually or semi-automatically segmented. More recently, artificial intelligence (AI) can automatically segment pediatric solid tumors. For preoperative planning, 3D models improve anatomic understanding and help multidisciplinary communication. During surgery, these models can assist the surgeon in accurately localizing tumors and determining resection margins in preclinical research settings. In patient education, 3D models present an opportunity to improve patient understanding of disease, therapy, and surgical risks. Lastly, digital 3D models are used in surgical training, particularly for challenging cases. Although digital 3D technologies are evaluated using technical performance metrics, these measures do not necessarily result in improved clinical outcomes. Consequently, evidence demonstrating improved surgical outcomes remains limited, also due to the small patient cohorts. Several challenges continue to hinder clinical translation, including image quality and standardization, segmentation complexity, organ deformability during surgery, and insufficient clinical validation. Addressing these limitations will require progress in four key areas: AI integration, multicenter validation, the development of centralized infrastructures including digital twins, and scalable implementation strategies that support widespread clinical adoption.

## Simple Summary

Digital three-dimensional (3D) visualization is an emerging adjuvant in surgery for children with cancer that addresses challenges associated with difficult anatomy and accurate removal of the tumor. Visualization with digital 3D models supports the planning of operations, guidance during surgery, explanation to patients and/or families, and surgical training by improving anatomical understanding, multidisciplinary communication, and surgical decision-making. Traditionally, 3D models rely on manual segmentation; however, artificial intelligence (AI) increasingly enables automated segmentation of solid tumors in children such as Wilms tumor and neuroblastoma. During surgery, 3D models may assist in the finding of a tumor and margin assessment, though most applications remain in preclinical testing. In addition, these models can enhance patient and parent understanding of disease and treatment and provide valuable training opportunities for complex procedures. Despite promising technical performance, evidence demonstrating improved clinical outcomes remains limited because of small patient cohorts with children with cancer and insufficient proof of actual benefit. Future work in this field depends on AI integration, multicenter validation studies, centralized infrastructures, and scalable implementation to make sure this technique is used more widely in the surgical field.

## 1. Introduction

Surgery remains a cornerstone in the treatment of children with solid tumors, especially for local control [1]. Surgical procedures have inherent risks such as incomplete resections, bleeding, damage to healthy tissues, and postoperative complications that impact recovery, adjuvant oncologic treatment, and long-term quality of life for children with cancer [2]. Moreover, surgical outcomes are dependent on the experience and volume of the operating surgeon and the treatment center [3]. This is particularly true in pediatric surgical oncology, where surgeons must navigate small anatomical structures and spaces, rare tumor types, and anatomical deviations with limited case volumes.

To optimize pediatric surgical and oncological outcomes, multiple innovative techniques are being explored that help the surgeon visualize the tumor and the adjacent normal anatomic structures. Three-dimensional (3D) guided surgery can be used as a complementary technique to standard cross-sectional 2D images to provide a 3D view of the tumor and relevant anatomic structures [4]. Three-dimensional models are created from medical imaging, such as computed tomography (CT) scans or magnetic resonance imaging (MRI) with segmentation. Segmentation is the delineation of various structures such as tumors, vessels, organs, soft tissues, and bones in medical imaging. Through various segmentation techniques, these structures are reconstructed into a patient-specific 3D model, in a digital or printed format [5]. Digital 3D models can be updated, distributed across platforms and accessed and displayed on various devices, including computers, phones and emerging augmented reality (AR) or virtual reality (VR) systems through head mounted displays. This enables surgeons to visualize relevant information and allows integration into surgical environments.

The current field of digital 3D modeling within pediatric surgical oncology is broad. Three-dimensional models are used in various applications including preoperative planning, surgical guidance, determining solid tumor resection margins and patient/family education, and surgical training [6,7,8,9]. In adult surgery, 3D models have become an integral part of standard surgical practice, particularly in orthopedic and oncologic surgery [10]. The use of 3D models in pediatric surgical oncology is still limited. Pediatric cancer is extremely rare, leading to small cohorts of patients that hinder large-scale clinical validation and the development of standardized workflows for digital 3D models for different purposes. Furthermore, segmentation is often technically challenging due to complex anatomy and heterogenous tumor location, which requires specific expertise, time, and additional resources, restricting broad clinical adoption. However, digital 3D models also present an opportunity to improve both surgical and oncological outcomes within the field of pediatric surgical oncology.

Therefore, in this white paper, we provide a comprehensive overview of the current landscape (Figure 1) of digital 3D modeling in pediatric surgical oncology. We explain the technical principles of 3D modeling and its clinical applications, including preoperative planning, intraoperative guidance, and patient/family education. We also highlight methods to validate these applications and determine the clinical impact. Additionally, we discuss existing challenges in implementation and validation. Also, we propose strategies to facilitate clinical translation and guide future directions in this evolving field.

## 2. Methods

This white paper was composed by an international multidisciplinary panel with expertise in pediatric surgical oncology, radiology, image processing, surgical navigation, and surgical simulation. The objective was to provide an overview of digital 3D technology in pediatric surgical oncology, identify barriers to clinical implementation, and define strategic priorities for future development and adoption within this field. The manuscript was developed through iterative online discussions and an in-person symposium (“New dimensions: imaging in pediatric surgical oncology”, June 2025, St. Jude Children’s Research Hospital, Memphis, TN, USA) to identify the current state of digital 3D technologies within pediatric surgical oncology. Additionally, the authors performed a targeted nonsystematic literature search to add to the descriptions of technologies, applications, perspectives, and recommendations. The search was conducted using PubMed and Google Scholar and included publications until June 2026. Search terms were structured around the main themes determined by the expert group, which involves 3D modeling, image segmentation, preoperative planning, intraoperative guidance, and surgical simulation. Publications were selected based on relevance to pediatric surgical oncology and their contribution to advancing digital 3D technologies in surgical care. As the search was intended to support descriptions of this white paper, no systematic screening or risk-of-bias assessment was performed.

## 3. Technology

### 3.1. Three-Dimensional Modeling

Patient-specific digital 3D models are created based on volumetric medical imaging datasets, most commonly CT scans, and MRI scans may be used for soft tissues and organs. The spatial resolution and quality of these images are crucial for accurate 3D modeling [11]. High spatial resolution, thin slice thickness, and isotropic voxels are necessary to ensure high fidelity of the anatomical 3D model. If the slice thickness is too large, especially in children, important details can be missed. Moreover, imaging contrast is essential for distinguishing specific structures. These radiological prerequisites can be difficult to achieve in very young patients.

A 3D model is created through image segmentation, where individual voxels are labeled to represent specific anatomical tissues, such as tumor tissue, solid organs, and blood vessels. Therefore, various open-source image segmentation software programs exist, including 3DSlicer (v5.12.4; The Slicer Community, Boston, MA, USA) and ITK-SNAP (v4.4.0; Penn Image Computing and Science Laboratory, University of Pennsylvania, Philadelphia, PA, USA) and commercial medical certified packages such as Mimics Innovation Suite (v28.0; Materialise NV, Leuven, Belgium) [12,13]. Within these software applications, several segmentation techniques are available. The most basic method is manual segmentation, annotating anatomical structures slice by slice. Another method is the use of semi-automatic segmentation, such as thresholding by selecting specific voxels within a defined range of intensity values, or using region growing, an algorithm that determines if a pixel’s values are comparable to neighboring pixels. Fortunately, in recent years, automatic segmentation algorithms have emerged with artificial intelligence (AI). These systems predominantly consist of deep learning algorithms [14]. They are trained on large imaging datasets with corresponding ground truth labels. The current state of the art is nnUNet, an open-source framework that is a self-configuration deep learning network handling preprocessing, network architecture selection, training, and post-processing automatically [15]. While it requires labeled ground truth annotations, these models can be easily trained for pediatric tumors. In adults, the focus has shifted towards foundation models [16] that are pre-trained on a vast and diverse collection of multi-labeled medical images, eliminating the need for additional training and enabling effortless segmentation of anatomical structures. However, foundation models have not been implemented yet in pediatric surgical oncology. It is not well understood how these foundation models work on small cohorts outside of their training data.

### 3.2. Visualization Technologies

#### 3.2.1. Displays, Stereoscopic and Autostereoscopic Display

Traditionally, the depiction of a 3D structure on a two-dimensional display (2D) uses rendering techniques: light, shade, and shadow. They are limited by fixed perspective and depth ambiguity. Two additional principles must be implemented to overcome these limitations: stereoscopy and motion parallax. Stereoscopy produces a sense of depth and solidity through two slightly different perspectives. Parallax describes the phenomenon whereby the relative positions of objects appear to change as one object moves around a landscape. Three-Dimensional displays, unlike 2D renderings, incorporate stereoscopy and parallax. The two varieties of display technologies incorporating stereoscopy and parallax include head mounted displays (HMD) and free-standing displays. HMD includes Virtual Reality (VR) and Augmented Reality (AR) headsets, while free-standing 3D displays include multi-view flat panels and volumetric displays.

#### 3.2.2. Augmented Reality and Virtual Reality

AR and VR technologies are increasingly used in surgical practice, education and training. Within surgery, AR can visualize 3D models directly on the patient or operating field. This allows for the interpretation of both patient tissue and virtually transparent anatomical 3D models at the same time. This capability allows surgeons to identify structures that may otherwise be obscured or difficult to distinguish.

Currently, two types of AR HMD devices exist: Optical See-Through (OST) and Video Pass-Through (VST) AR systems. OST displays are transparent, allowing surgeons to view the real world while simultaneously projecting digital information in the vision of the surgeon. In contrast, AR VST uses outfacing cameras to capture the real world, and the surgeon perceives the real world through a virtual display.

Unlike AR, where a user can still see the real world, VR immerses users in a fully virtual environment. This allows the user to be completely disclosed from the world, which makes it especially useful for viewing 3D models for patient education or surgical simulations and training.

### 3.3. Navigation Technologies

For surgical navigation and guidance, 3D models can be utilized by establishing a connection between a digital environment and the intraoperative field. In this context, 3D models of anatomical structures or surgical instruments are also presented in a digital environment and are continuously updated based on real-world events. Two fundamental principles are essential to achieve this: registration and tracking. Registration is necessary to align the digital 3D model with the real-world spatial location of a specific organ or tool, while tracking ensures that the position of the 3D model is continuously updated, even in the presence of tissue deformation or movement. Multiple modalities and techniques exist to achieve accurate registration and tracking.

#### 3.3.1. Augmented Reality

AR can be utilized to visualize digital 3D models; however, AR can also be employed for surgical navigation and guidance. In this context, registration and tracking are achieved with the integrated outward-facing sensors that monitor the surgical environment. These sensors are often RGB cameras or Infrared sensors. Additionally, 3D point clouds are utilized to determine the geometric correlation between the preoperative digital 3D model and the surgical environment, enabling registration. Recent advances in AR have focused on deep learning algorithms for the automatic registration of 3D models based on RGB video data from the HMD to superimpose the 3D model onto the operative field. Similar techniques have been validated in laparoscopic surgery for adults, although this field is still in its early stages as problems such as occlusion or partial scenes need to be addressed [17].

#### 3.3.2. Electromagnetic Navigation

Electromagnetic (EM) tracking systems use a local magnetic field that allows the tracking of surgical tools and sensors, and which position and orientation can be visualized in a digital 3D environment. When combined, accurate position and tracking enable surgical guidance without problems in line-of-sight, which is particularly relevant for small children. EM sensors consist of small coils in a magnetic field to determine their relative position and orientation [18]. Due to the small size of these coils, it is possible to integrate these sensors within surgical tools or fixate sensors on organs or tissues. Tracking surgical tools with EM, such as tracked intraoperative ultrasound, it becomes possible to perform registration on bones or other anatomical structures near the tumor. In case of tracked ultrasound, the surgeon performs an ultrasound sweep on a reference bone structure, and an algorithm automatically detects the bone surface and 3D geometry. Then, the preoperative digital 3D model of the bone can be connected to the intraoperative situation based on the ultrasound sweep, enabling 3D surgical guidance.

### 3.4. Training/Simulation Technology

Digital 3D technologies can support surgical simulation through several complementary platforms, ranging from fully virtual environments to hybrid physical-digital models. In pediatric surgical oncology, these technologies are particularly relevant because complex tumor resections are rare, anatomically variable, and associated with high-stakes intraoperative decision-making.

VR provides a completely digital environment in which surgeons can explore patient-specific anatomy, rehearse operative approaches, and develop spatial understanding before entering the operating room. Software covering surgical simulation and dissection, patient assessment and triage, radiology and emergency care, among others are available for purchase. However, despite growing interest in simulation-based surgical education, relatively few patient-specific simulation models have been developed specifically for pediatric surgical oncology. Advanced patient-specific virtual surgical simulation platforms can incorporate deformable biomechanical tissue models allowing users to manipulate virtual instruments and rehearse the entire procedure on a patient-specific model for pediatric oncology [19]. However, current VR systems remain limited in their ability to reproduce realistic tissue handling, traction, bleeding, and tactile feedback, and lack the physical connection to the patient that tangible 3D models provide.

AR and Mixed Reality (MR) allow digital 3D models to be visualized within the real-world environment. For training purposes, these technologies can be used to overlay tumors, vascular, and organ anatomy onto physical phantoms, mannequins, or operative simulations to create a hybrid physical-digital training model.

## 4. Applications

### 4.1. Preoperative Surgical Planning

The initial use of patient-specific 3D models in pediatric surgical oncology was its application in preoperative planning [20,21,22,23,24]. The 3D visualization allows surgeons to obtain an integral perspective of the anatomical and pathological relationships relevant to the upcoming surgery [25]. Three-Dimensional models visualize tumor location, volume, and relationships to critical structures. Typical use cases include abdominal and thoracic neuroblastoma, renal tumors for nephron-sparing surgery (see Figure 2, left panel), liver tumor surgery, sacrococcygeal teratomas, and Ewing- and rhabdomyosarcomas at specific localizations [26,27]. Also, 3D models can be insightful for atypical localizations, very rare diseases, or to model biological markers of the tumor and heterogeneity [28]. Noteworthy, it is important to define the expected clinical use of a 3D model beforehand. This is crucial for the developer to decide what will be relevant in the model. It also decides the visualization approach; 2D monitor, 3D print or a head mounted display.

Beyond conveying spatial information, 3D models can provide a framework for approaching a surgical case, even when information is ambiguous. Not infrequently, the 3D image processing specialist and radiologist may struggle to segment a portion of a mass, unable to distinguish tumor from post-treatment change. During pre-operative planning sessions, these uncertainties can be discussed, providing anticipatory guidance to the surgeon. At post-op debriefings, the surgeons render their final judgement on the accuracy of the models. Similarly, the use of 3D models can facilitate discussion of complicated cases at multidisciplinary tumor boards, clinical care meetings, and educational conferences. An overview of literature for all applications is given in Table 1.

### 4.2. Intraoperative Guidance

#### 4.2.1. Augmented Reality

Current techniques of AR within pediatric surgical oncology have at present limited standard clinical applications but are explored in research settings. AR for the localization of chest wall tumors has been developed and validated by van der Woude et al. for intraoperative use [29]. It showed potential to eliminate more invasive localization methods, but at that time only feasibility was studied. A recent study by de Groot et al. used a similar system to assess the feasibility of using 3D holograms for Wilms tumors in patients undergoing a total nephrectomy [30]. Although the study design focused on total nephrectomy as to not impact surgical decision-making, the AR application is intended to be utilized during nephron sparing surgery (NSS) to assist a surgeon in determining the resection margins. This landmark-based AR approach showed poor results due to difficulties in landmark registration in a small pediatric abdomen and due to deformability of the target organ. Another retrospective study by Bronowicki et al. compared outcomes of pediatric surgical procedures with and without MR based on hospitalization and operation times. They included a wide variety of pediatric surgical oncology procedures, including thoracotomy, biopsies, and diverse tumor resections. While their sample size was small and some cases had longer operative times, their findings suggest that MR did not significantly prolong operative times or hospital stays [31]. Although current AR applications remain largely, they hold promise for intuitive and flexible integration into pediatric surgical oncology practice. However, further technical development is required to improve accuracy, robustness, and real-time adaptation to intraoperative anatomical changes before routine clinical implementation and larger clinical trials can be achieved.

#### 4.2.2. Electromagnetic Tracking

Electromagnetic tracking for open surgery is considered fast, easy to use, allows for reregistration, requires no radiation and does not have problems with line of sight. This technique has already been used in open surgery for adults, with diseases such as liver metastases and lymph nodes [32,33,34]. The surgeon can see the location of the actual surgical tool in reference to the digital 3D model on a 2D screen. This allows for direct navigation towards a surgical target. Because the physical and digital world are now coupled, these systems also allow for safety warnings or advanced algorithms to assist the surgeon and increase patient safety. Figure 3 gives a surgeon view of electromagnetic navigation used on a phantom to localize lymph nodes in the pelvis.

In the context of pediatric surgical oncology, such systems may be used for many different applications, both for determining localization and margins, in the context of neuroblastoma, Ewing sarcoma, lymph node removal, or NSS [35], but this first requires clinical validation studies. The system has not yet been used in pediatric surgical oncology, and a first-in-child study is expected to start soon (Study name: ORCAS—Oncologic Resection of Complex Pediatric tumors with Accurate Navigated Surgery; Dutch CCMO ID: NL-OMON61296).

### 4.3. Patient and Family Education

Patient- and family-centered communication and education are essential components of high-quality pediatric cancer care and informed consent. Research has shown that most pediatric patients and parents want to understand details about their illness, including treatment options, risks, and benefits [36]. Recent technological advances in VR offer new opportunities for patient-centered communication, education, and informed consent procedures in the setting of pediatric cancer [37].

At St. Jude Children’s Research Hospital, more than 60 VR sessions have been conducted with family members of patients undergoing surgical resection of soft tissue tumors of the chest and abdomen (unpublished data). During these sessions, 3D models were virtually viewed by both the surgeon and family members, engaging in real-time discussion of the proposed surgical intervention, risks, and benefits. Post-VR data collected from family members who viewed 3D images of their child’s disease reveal the emotional and psychological impact of this intervention: “I wish I had seen this sooner;” “He has been complaining of pain in all these areas, and I just now realize the disease size;” “Now I understand why they sent us here for surgery;” “I don’t think I understood how bad it was before;” “It is so much more real.”

### 4.4. Surgical Training

Surgical oncology training models are valuable educational tools derived from digital 3D models, which accurately reproduce highly specific and complex scenarios, particularly in pediatric surgical oncology, where training opportunities are limited by the low case volume and rarity of many tumor resections. Recent virtual surgical simulation systems have shown promise for improving surgical evaluation and training in complex pediatric liver tumors [19].

Although patient-specific physical 3D simulators fall outside the primary scope, they provide a valuable complementary perspective by demonstrating how patient-specific 3D physical simulators can be translated into hands-on surgical rehearsal, while adding tactile feedback and tissue interaction that current virtual environments cannot yet fully replicate. Within this context, physical simulators in pediatric surgical oncology may provide clinical value by enhancing the surgical team’s preoperative preparation [38]. They enable a more accurate and intuitive understanding of the spatial relationships between tumors, vascular structures, and adjacent organs, thereby potentially improving surgical planning and decision-making, especially in the context of nephron-sparing surgery or neuroblastoma [39,40]. The realistic proportions and geometry closely replicate intraoperative anatomy [41,42]. The tactile component, which is difficult to simulate in a virtual environment, of the simulator allows teams to hands-on experience, facilitating a better appreciation of surgical planes, helps anticipate technical challenges, and promotes alignment of operative strategies in a more concrete and collaborative way than imaging alone. This may improve surgical precision, the risk of injury to critical structures may be reduced, and overall procedural safety may be enhanced.

**Table 1:** This overview presents a selection of studies for each application. It highlights the demonstrated technical and clinical benefits, the maturity of development following the IDEAL framework, Clinical maturity, and the corresponding level of clinical evidence through the OCEBM levels. It also illustrates the gap between technical development and current clinical evidence.

3D Applications	Author	Type of Study	Main Demonstrated Benefit	Clinical Outcome	IDEAL Stage *	Clinical Maturity	Level of Evidence (OCEBM) **
Preoperative Planning	Chaussy et al. [21]	Technical validation	Improved anatomical understanding, surgical planning and multidisciplinary communication	Limited evidence for improved surgical outcomes & reduced positive margins	2b–3	Established	Level III–IV
	Valls-Esteve et al. [23]	Case series					
	Fitski et al. [43]	Retrospective cohort					
Intraoperative guidance (AR/MR/EM)	Van der Woude et al. [29]	Clinical Feasibility study	Improved tumor localization and margin visualization	Not yet proven clinical benefit	2a–2b	Development and first in-pediatric studies	Level IV
	De Groot et al. [30]	Clinical Feasibility study					
	Bronowicki et al. [31]	Case series					
	Van Boheemen et al. [35]	Phantom study					
Patient/Family Education	Tejo-Otero et al. [36]	Observational studies	Improved understanding, communication and engagement	Not yet evidence for long term clinical or psychosocial outcomes	2b	Established	Level IV
	Robertson et al. [37]	Narrative Review					
Surgical Training	Valls-Esteve et al. [44]	Technical study	Improved procedural understanding and training realism	No evidence for improved operative performance or patient outcomes	2a–2b	Development and first testing	Level IV
	Fitski et al. [39]	Feasibility study					
	Xiu et al. [19]	Simulation study					

* IDEAL Stage. 0: Preclinical, 1: Idea, 2a: Development, 2b: Exploration, 3: Assessment, 4: Long-term study [45]. ** Level of Evidence: Classified according to the Oxford Centre for Evidence-Based Medicine (OCEBM). Level 1: systematic reviews or randomized control trials, Level II = individual RCT’s, Level III: non RCT or follow up studies, Level IV: case series, observational studies, Level V: reasoning or expert opinion [46].

## 5. Validation and Clinical Impact

### 5.1. Three-Dimensional Model Accuracy

The global accuracy of segmentation techniques and their resulting 3D models are primarily validated through well-known technical metrics, in particular the Dice coefficient and Hausdorff distance [47]. The dice coefficient measures the similarity between the ground truth label and the predicted label, while the Hausdorff distance represents the maximum distance between any point on the ground truth label and the nearest point of the predicted label. A high dice score of a tumor model will have a seemingly high fidelity, but this does not reflect the accuracy of the relationship between the tumor and adjacent organs. This is often considered more relevant for the surgeon. The same holds true for the Hausdorff distance. If the outlier is far away from crucial structures, it is not necessarily relevant to the surgeon. Moreover, metrics are based on ground truth segmentation. Ground truths are typically provided by a radiologist or an experienced developer but can also show a large interobserver variation [48]. This variation may even increase after preoperative chemotherapy, as it causes more tumor heterogeneity. Technical image-analysis metrics give a global indicator of the accuracy of a 3D model and do not directly relate to clinical value and local accuracy of the model. It is recommended for surgeons and developers to always validate the local accuracy of the 3D model with corresponding medical imaging and a trained medical specialist. Such nuances of the accuracy of the technique should always be studied to make an accurate preoperative plan and crucial intraoperative decisions.

### 5.2. Surgical Navigation Accuracy

In the context of surgical navigation, we aim to determine whether a 3D model used for intraoperative surgical guidance correctly overlaps with the actual surgical environment. Multiple technical evaluation metrics can be used, of which Target Registration Error (TRE) is the most clinically relevant and often used within research [49]. It is defined as Root Mean Square error of the distance between the true location of a target point and a target point shown in the visualization after registration. This error indicates the extent to which the digital 3D models correspond to the actual situation, which is important for the surgeon to know. Each procedure has its own required maximum TRE, as this depends on the anatomy and surgical need. However, a low error does not necessarily correlate with matching anatomy. Firstly, to truly determine the accuracy of the surgical navigation system, a surgeon must compare a landmark location in the digital scene with the intraoperative field. This can be challenging due to the deformability of the organs in the surgical field, while the digital scene remains solid and rigidly aligned. These deformability errors are difficult to determine quantitively. Secondly, a slight rotation error can lead to increased inaccuracy further away from the rotating axis, due to the lever effect.

### 5.3. Usability and Cognitive Load

Digital 3D models demonstrate value in the ability of the surgeon to locate specific anatomy. Yet it is crucial to validate the impact of the system on the surgeon and the surgical workflow to determine its value within the clinical practice. This is particularly relevant as the introduction of new devices also causes additional risks. Surgeons must acquire the necessary skills to use these techniques safely, which can be made easier by enhancing the usability of these systems, which could facilitate their clinical integration. Device usability is primarily assessed using standardized instruments like the System Usability Scale and ISO 9241-11 criteria [50]. Cognitive workload can be evaluated using NASA-Task Load Index or the surgical adaptation named SURG-TLX [51,52]. These measurements aim to assess the influence of the system on the surgeon, as a metric for surgical performance and correlated clinical outcomes. However, these metrics are qualitative, and currently there is not a specific quantitative measurement that determines the cognitive load or stress levels of a surgeon during the use of a device. In the recent literature, physiological parameters have been used to determine stress levels during procedures, which could be used to validate whether a medical device has influence on stress, but this has not been extensively explored within surgery and is often prone to other factors such as experience or other activities [53].

### 5.4. Implementation Phases

The implementation of these surgical technologies necessitates thorough validation across predefined phases. Existing guidelines, such as the IDEAL framework, provide a structured approach to validating these technologies [45,54]. The IDEAL framework consists of the phases of idea, development, exploration, assessment, and long-term study. For the development phase, surgical simulators could play an important role in determining the usefulness of surgical applications in an early phase. Although surgical simulators provide a clear basic qualification of the technique, there always remains a mismatch to the real world due to differences in deformation, abdominal anatomy, and the surgical working environment [39,55]. Consequently, conducting clinical feasibility studies remains crucial, as these studies can identify limitations that are not possible to replicate during phantom experiments.

### 5.5. Clinical Outcome and Decision Making

Studies based on digital 3D models in preoperative surgical planning in pediatric surgical oncology have only shown limited evidence of improvements in clinical outcomes. A recent retrospective single center cohort study showed a reduced amount of positive surgical margins in patients undergoing nephron-sparing surgery [43]. However, these improvements could not be allocated to the use of 3D models alone, but also to other factors including concurrent centralization of care and the implementation of intraoperative ultrasound. Others have reported improved surgical outcomes in pediatric liver surgery and have reported case series with 3D models for very complex cases such as pelvic tumor surgery [40,44,56]. Unfortunately, this is still only circumstantial evidence, and there is no hard proof of improved clinical outcomes.

The effects on clinical impact are very difficult to study. Retrospective analyses after implementation with a matched cohort are feasible. However, these analyses contain variation in patients and many confounders. Prospective studies take a long period of time due to small patient cohorts. During this period, the techniques develop further at a much faster rate leading to either variation in the used technique or a pause in development, limiting the evidence or clinical value of the evidence. This results in a lack of direct clinical evidence for the use of digital 3D technology. Currently, there is a prospective multicenter randomized controlled trial running aiming to overcome these problems in the context of 3D models for neuroblastoma surgery. This study is performed by Sant Joan de Déu hospital in Barcelona leading to real-world evidence of clinical impact (Study name: Use of Virtual Reality in Surgical Planning for Neuroblastoma; ClinicalTrials.gov ID: NCT05781919). More high-level clinical evidence is necessary among more different clinical indications.

Nevertheless, proof of 3D modeling for preoperative planning may not necessarily be required. The technique is versatile, easily implemented, and can be applied with moderate costs. If the surgical team can rely on the 3D model and perceive benefit from the technique, 3D models can be implemented in surgical care. Evidence of clinical outcomes will then follow in the long run.

For intraoperative surgical navigation systems, there is also no clinical evidence yet in the field of pediatric surgical oncology. These technologies are still under development or undergoing clinical validation. Looking at the evidence in adult oncologic surgery, true clinical value is expected from these techniques. However, clinical evidence will have to be studied in multi-center prospective trials to ensure a high level of evidence in a significant number of patients in a timely manner.

### 5.6. Cost-Effectiveness and Scalability

The cost-effectiveness of digital 3D models in pediatric surgery remains unclear and has not been extensively studied. This is primarily due to the challenges in determining the costs and effectiveness of digital 3D models for various clinical applications. Creating digital 3D models involves personnel, software, and hardware expenses. Segmentation requires manual input and evaluation from trained professionals before clinical use. Costs vary per indication, with more detail required for complex cases like retroperitoneal neuroblastoma with vessel encasement increasing costs, in comparison to easier cases such as nephron-sparing surgery. Additional software and/or hardware may be needed depending on the application. For surgical navigation, additional hardware or assistance during the OR is required. As mentioned earlier, the improvement of clinical outcomes is also not scientifically clear. The clinical outcomes and associated improvements in QALYs also depend on the application such as surgical navigation or patient education. Especially in the case of 3D digital models not directly altering surgical treatment, such as patient education, it will be difficult to determine the long-term effectiveness. These varying fixed and variable costs and lack of understanding of effectiveness make it challenging to determine the true cost-effectiveness of these techniques. However, startup costs are relatively low, and individual centers often decide it is worth the initial investment after pilot studies. In the future, delivering 3D models may need to be centralized to further reduce costs per patient and automated process, and the integration of AI could improve scalability of the techniques. Secondly, cost-effectiveness and health assessment studies should be conducted after techniques have scientifically demonstrated clinical efficacy. It is likely that 3D modeling will first show cost-effectiveness in complex cases, where the overall cost of care is high and therefore, the potential for significant savings relative to the fixed cost of modeling is also high.

## 6. Knowledge Gaps and Future Directions

Based on this white paper, we have defined explicit clinical and technological knowledge gaps. These gaps should be addressed in future research. Moreover, we also define research priorities.

### 6.1. Clinical and Technical Knowledge Gaps

#### 6.1.1. Digital 3D Technology for Laparoscopic Video

While laparoscopic surgery has become more accessible in the field of pediatric surgical oncology, digital 3D technology is not yet actively studied in this clinical context. A digital 3D model may be used for preoperative planning of laparoscopic surgery, as for any other case. The 2D camera view, different zooming levels and intricate anatomical relationships and angles in the surgical cavity make the translation from 3D model to an intraoperative view more difficult. No applications of intraoperative 3D modeling during laparoscopy have been described in the literature. However, the digital video stream of the laparoscopic camera is a key available component for computer vision techniques for surgical navigation. No additional hardware such as AR and electromagnetic tracking may be necessary to implement digital 3D models in the intraoperative view. Such techniques are already becoming more accessible in adult oncologic surgery but are absent from pediatric surgical oncology. In robotic surgery, some 3D consoles even allow direct intraoperative projection of the 3D model, but robotic surgery remains very limited in pediatric surgical oncology. Thus, this knowledge gap may be addressed in the future.

#### 6.1.2. AI Integration

Current work on the technical development of digital 3D models is primarily in the field of AI segmentation. These algorithms can automatically compute the segmentation of the anatomy and pathology. This removes interobserver variability and reduces the development time significantly. AI-algorithms are typically specific per pathology or organ and medical imaging technique. New approaches, so-called self-configuration or foundation algorithms, combine multiple AI-algorithms to compute different segmentations with one algorithm, e.g., nnInteractive, nnUNet and TotalSegmentator [15,57,58]. Future applications will integrate these algorithms, possibly combined with pathology-specific algorithms, to create 3D models with much reduced development times. Yet it remains crucial to control the local accuracy of the output of such algorithms and resulting models before using it as a surgical planning tool in pediatric surgical oncology. Specifically, when implementing AI algorithms, it is crucial to perform post-implementation surveillance to detect drift of the performance of the algorithm [59]. Also, 3D Labs should be aware of the local medical device regulations when using these technologies outside of in-house research studies. Clinical applications require full compliance with medical device regulations as these technologies are considered software as a Medical Device.

#### 6.1.3. Digital Twins

Digital twins integrate all described digital technologies and multimodal clinical data with postoperative outcomes to improve the predictability of surgery and improve intraoperative decision making [60,61]. They simulate surgical scenarios by dynamically updating its outcome prediction based on the currently available spatial and temporal information of the patient. With this patient data, these virtual constructs could enable more precise surgery, risk stratification, and surgical workflow optimization [60]. A digital 3D model plays a crucial role within digital twins, as they visualize outcome prediction for the surgeon. Digital twins, currently developed for adult surgery, have the potential to significantly impact pediatric surgical oncology as we approach each unique case. With the relatively low number of patients, digital twins offer a virtual model to determine the best surgical treatment for the individual patient without direct clinical consequences [62]. However, the low number of patients for the digital twin will also be the major hurdle. Digital twins rely on vast amounts of heterogenous data to accurately predict the optimal surgical pathway, much more than the currently used data for digital 3D models. Obtaining data but also validating the predicted outcome will be a major challenge in our field. Therefore, research groups working on the transition from digital 3D models to digital twins should prioritize international validation and development. Digital twins will then enable a new more validated approach for digital 3D precision surgery in pediatric surgical oncology.

#### 6.1.4. Hybrid Simulation Models for Surgical Training

Virtual simulators, as mentioned earlier, have been insufficiently explored within pediatric oncological training. However, advancements in virtual simulators have improved their ability to simulate tactile feedback. By combining virtual simulators with AR or MR, digital 3D models can be visualized within the real-world environment, providing a more immersive and realistic training experience. For training purposes, these technologies can be used to overlay tumors, vascular, and organ anatomy onto physical phantoms, mannequins, or operative simulations to create a hybrid physical-digital training model. These hybrid simulation models allow hands-on rehearsal of critical steps such as parenchymal-sparing dissection and vascular exposure while safely guided with AR. Communication between surgeons working on a hybrid simulation model may be improved in comparison to VR alone. Ultimately, both advanced virtual simulators and hybrid simulation models have the potential to improve the accessibility of patient-specific surgical training by allowing repeated rehearsal of rare and complex pediatric oncologic procedures outside the operating room, thereby expanding training opportunities beyond the limited number of clinical cases available.

### 6.2. Future Directions

#### 6.2.1. Centralization

Making 3D techniques more broadly available for other surgeons who do not have 3D facilities is a major challenge. Not all hospitals have access to this technology and resources to start 3D initiatives. The centralization of 3D care through international 3D Labs may overcome this challenge. An example comes from the current multicenter 3D trial led by Sant Joan de Déu hospital in Barcelona. They have been able to include many international centers and provide 3D models for many patients within this prospective trial. Centralization of 3D modeling also increases the work volume of the specific center significantly. This can reduce the costs per model, increase local knowledge, quality and efficiency, improve scientific studies, and improve technical development. Moreover, with the relatively low number of patients in pediatric oncology, a geographical region may only need a few international 3D labs to be able to deliver 3D care across the region.

Beyond clinical implementation of 3D technology, international collaboration should be a research priority. The low incidence of pediatric cancers makes it challenging for individual centers to evaluate and refine emerging technologies such as 3D planning, patient-specific models and surgical navigation techniques. Unlike many traditional medical technologies, 3D innovations are often largely digital, enabling expertise, software, and virtual 3D models to be shared internationally with relative ease. Furthermore, because pediatric patients represent a relatively small market, new surgical technologies and medical devices are often developed and validated with intended use in adults, creating a risk that pediatric surgical oncology lags behind adult surgical care. International research networks can address these challenges by combining expertise, leveraging high-volume centers to drive innovation, and facilitating multicenter data sharing and validation. However, multi-center multi-country sharing of imaging data or technology raises concerns of data governance. Data sharing agreements and patient approval will have to be organized to ensure the local laws adhered to before centralization is fully implemented.

#### 6.2.2. Clinical Proof of Digital 3D Technology

There is currently no definite proof that digital 3D models have a significant impact on the surgical outcome of a child. Different groups have performed different analyses to prove the clinical benefit of digital 3D technology for preoperative planning, from case series to retrospective designs, but no RCT has been performed yet. Results of the first RCT (ID: NCT05781919) are expected. For intraoperative navigation, only feasibility studies and small case series have been performed and remain scarce. Yet the results from research groups working on this matter are highly anticipated. The expected clinical value of these technologies is significant in terms of surgical confidence, operating times, improved localization of tumors and metastases, more complete removals, and reduced complications. Digital 3D technology during surgical consultations with patients and caregivers is used more often but is also scarcely studied. It is inherently difficult to prove additional value of this technique in a multifactorial setting. There is no one-technique-fits-all approach when it comes to understanding and experienced stress. While, as addressed in the section “Clinical outcome and decision making”, definite proof may not be necessary for the technique to be implemented based on the expected value and costs, it remains crucial that we gather clinical evidence and perform well-structured studies on this topic. As evidence remains limited to lower levels of evidence and specific implementations in small research settings, a meta-analysis helps progression and evidence, although the certainty of the resulting evidence will remain limited by the methodological quality and risk of bias of the included studies.

## 7. Conclusions

Digital 3D technology has specific benefits in the field of pediatric surgical oncology. This technique can be used for surgical planning, patient education, surgical training, and intraoperative navigation. With current open-source approaches, it is relatively easy to implement during surgical planning. However, surgeons should be aware of the technical nuances for their clinical practice. True clinical benefit has not been directly proven, yet it is to be expected and currently studied in a multi-center international trial for neuroblastoma surgery. Intraoperative 3D techniques are currently under development specifically for children. Future work should focus on making digital 3D technology centrally available, improving modeling through image quality and AI, and integrating multimodal data with digital twins. These efforts in digital 3D modeling aim to improve the surgical outcome of children with cancer.

## Figures and Tables

**Figure 1:**
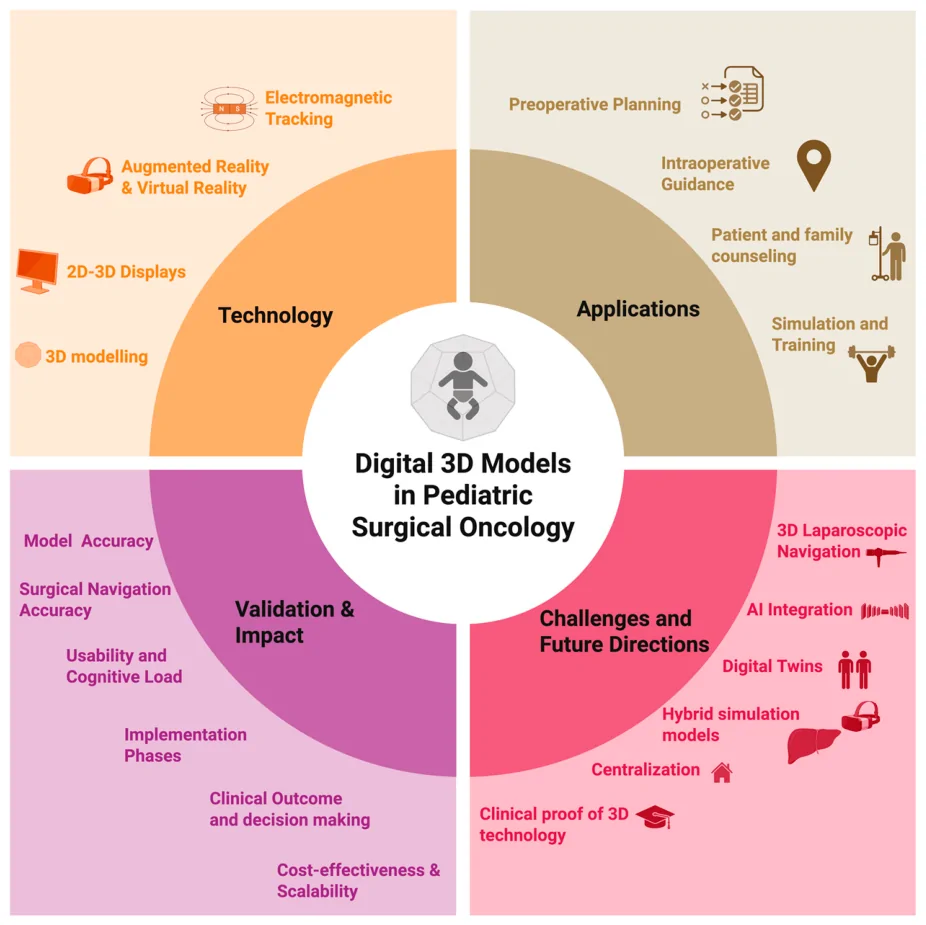
Overview of the current landscape of 3D modeling within pediatric surgical oncology.

**Figure 2:**
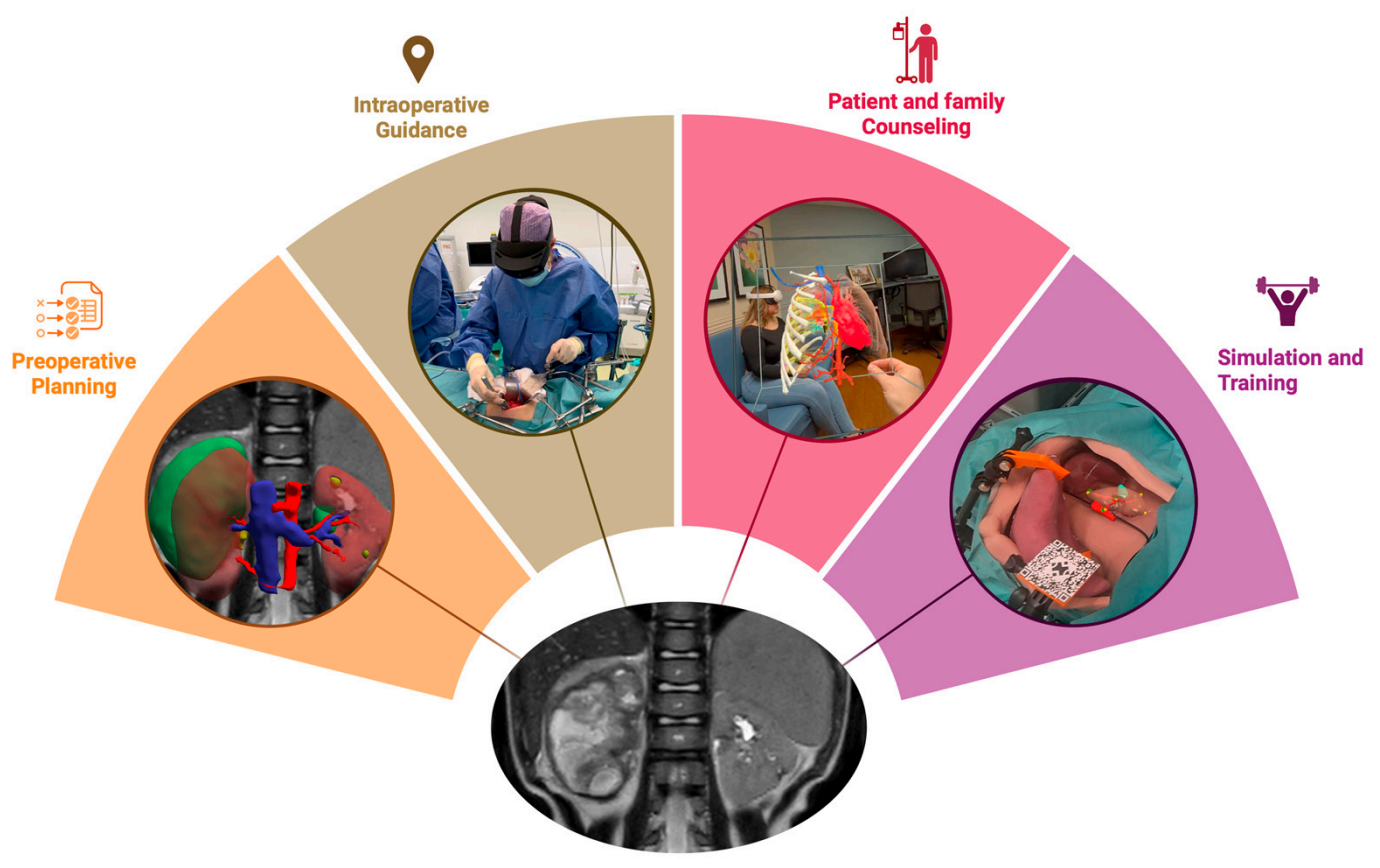
Overview of the applications of 3D digital models in pediatric surgical oncology all derived from the medical imaging (MRI).

**Figure 3:**
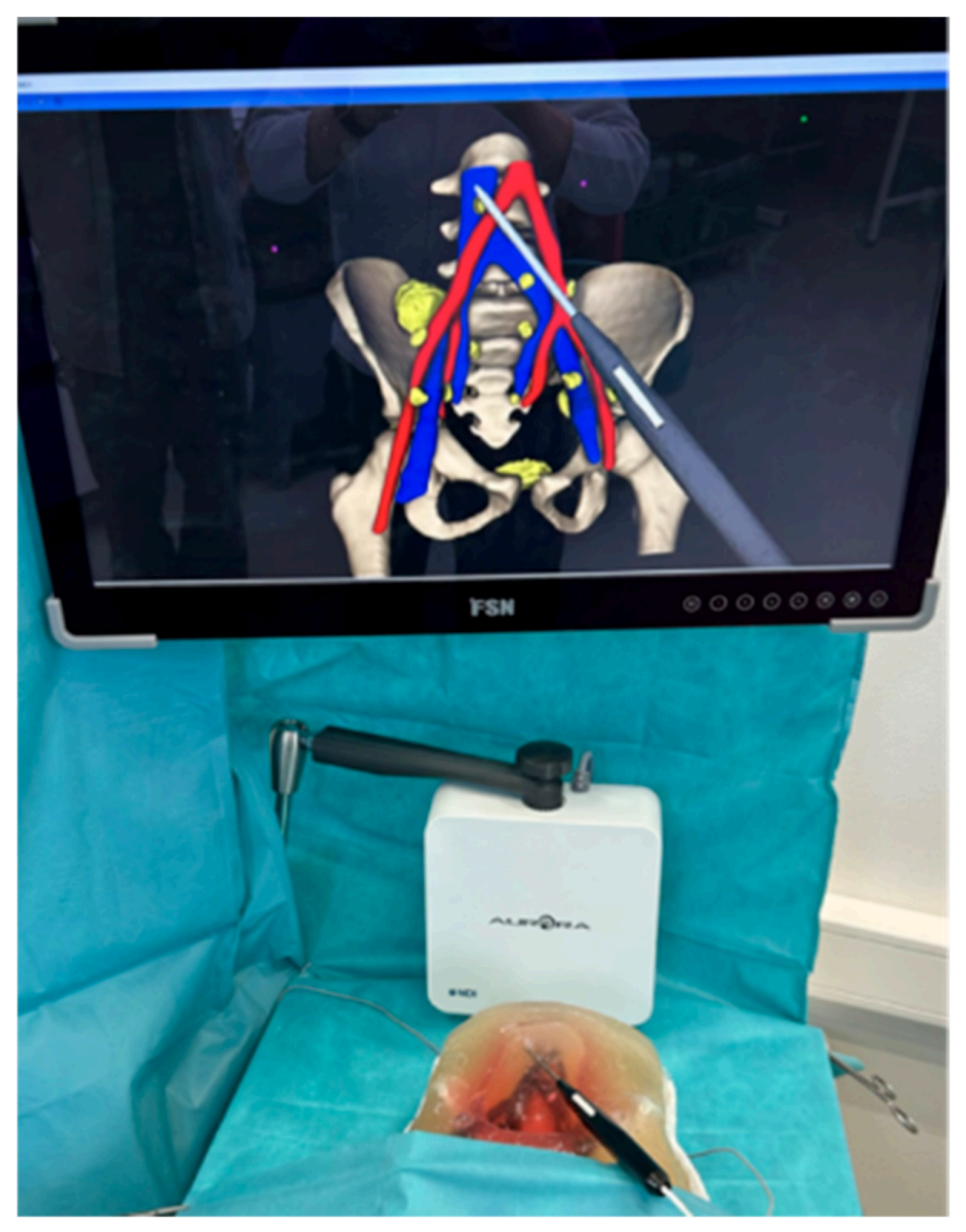
An overview of a surgical phantom setup of electromagnetic navigation used to find the lymph nodes in a pelvis. The electromagnetic field generator (NDI Aurora; Northern Digital Inc., Waterloo, ON, Canada) is behind the phantom and is positioned underneath the patient’s bed during surgery. The colors on the display represent the following anatomical structures: Red: arteries; Blue, veins; Yellow: simulated tumors/lymph nodes; Beige: bone.

## Data Availability

No new data was created or analyzed in this study. Data sharing is not applicable to this article.

## Abbreviations

The following abbreviations are used in this manuscript:

2D	Two-Dimensional
3D	Three-Dimensional
AI	Artificial Intelligence
AR	Augmented Reality
VR	Virtual Reality
EM	Electromagnetic
HMD	Head-Mounted Display
OST	Optical See-Through
VST	Video Pass-Through
NSS	Nephron Sparing Surgery
MR	Mixed Reality
TRE	Target Registration Error
